# Editorial: Advances in tissue repair and regeneration

**DOI:** 10.3389/fmedt.2022.1066486

**Published:** 2022-11-03

**Authors:** Woojung Shin, Hyun Jung Kim

**Affiliations:** ^1^ Wyss Institute for Biologically Inspired Engineering, Harvard University, Boston, MA, United States; ^2^ Institute for Medical Engineering and Science, Massachusetts Institute of Technology, Cambridge, MA, United States; ^3^ Department of Inflammation and Immunity, Lerner Research Institute, Cleveland Clinic, Cleveland, OH, United States

**Keywords:** regenerative medicine, tissue engineering, organ-on-a-chip, 3D bioprinting, organoid, biomaterials, biomechanics and mechanobiology, one health initiatives

**Editorial on the Research Topic** Advances in tissue repair and regeneration

Regenerative medicine and its associated technologies have evolved rapidly over the past couple of decades. Several representative examples include primary organoid culture, 3D bioprinting, novel biomaterials or hydrogel systems, and organ-on-a-chip. The development and advancements of the technologies have led to significant breakthroughs in various biomedical research including *in vitro* disease- and cellular- mechanistic studies, pharmaceutical drug screening and validation, *in vivo* implantation for cell-based therapies, and translational medicine between animals and humans. This Research Topic contains 2 research articles and 2 review articles that address the recent advances, significance, and perspectives in regenerative medicine and pertinent technologies to be potentially implementable in the management of tissue injury.

Trengove et al., introduced a new approach to enhancing the adhesion of gelatin methacryloyl (GelMA) hydrogel to cartilage tissue. The study demonstrated that the addition of microbial transglutaminase to GelMA, combined with photo-crosslinking, can enhance the mechanical properties of the hydrogel as well as the adhesion capabilities to *ex vivo* bovine or human cartilage. The new system was biocompatible and did not impact the metabolic activity of human adipose-derived stem cells encapsulated in hydrogel over 7 days of the culture period. Because one of the main challenges of clinical cartilage implants is integrating and retaining implanted materials within the surrounding native environment, this new method suggested a potential to improve the adhesion capabilities of GelMA-based implant technology.

DiCerbo et al. studied the mechanosensing properties of mesenchymal stem cells (MSCs) depending on the compression and porosity of the surrounding composite. The study utilized a polycaprolactone (PCL) scaffold and perfused MSC-laden methacrylated gelatin (GelMe) hydrogel in the scaffold. Using a computer simulation as well as a microscopic observation on the cell morphology and nuclear Yes-associated protein (YAP) signal, it was concluded that elastic hydrogel systems can apply different compression stress in different regions (i.e., edge vs. center) that can lead to locally different morphologies and nuclear YAP signals to MSCs. The cell morphology and nuclear YAP signal also varied depending on the porosity of the scaffold. These results will be useful in further mechanistic studies in stem cell mechanobiology and tissue engineering to develop better composites that can induce physiologically reliable differentiation of transplanted stem cells *in vivo*.

Han and Jang discussed the recent advances of *in vitro* gut models and the importance of developing reliable gut-brain axis (GBA) platforms to study neurological diseases. The article reviewed existing *in vitro* gut models with different anatomical features and the means to achieve them, such as microfluidics, patterning, scaffolding, and bioprinting. Finally, the review concluded that the right enteroendocrine model is necessary to reconstitute physiologically relevant microenvironmental cues using advanced techniques, such as bioprinting, to study the influence of GBA in neurological diseases. The authors addressed the critical points and the development and advancements of *in vitro* GBA models that encompass essential cell types will substantially enhance our understanding of mechanisms and therapeutic strategies of neurological diseases that are largely uncovered.

Kawasaki et al., reviewed the recent advances in developing animal organoids and their potential impact on better understanding human diseases to pursue One Health Initiatives. The article summarized the existing organoid models of farm and companion animals categorized by organ systems, then discussed the potential roles of animal organoid models in public health, food security, and comparative medicine. Finally, the review elaborated on the organoid culture platforms and the need to integrate animal organoid cultures into advanced culture platforms such as microfluidic organ-on-a-chip models for better reconstituting the physiological microenvironment. This review article provides new insights to biomedical scientists, veterinary scientists, and pharmaceutical investigators to explore the unique utility of organoid models derived from farm and companion animals for the One Health Initiatives.

The articles published on this Research Topic cover a broad spectrum of research in regenerative medicine and its technologies which will bring new insights and perspectives for future technologies. We would like to appreciate all the authors for their contributions to this Research Topic. It is also critical to acknowledge reviewers' contributions for ensuring the scientific validity and quality of the studies submitted to this issue.

